# Optimizing Informed Consent for Australian Newborn Bloodspot Screening and Research: Consensus Workshop Insights and Recommendations

**DOI:** 10.3390/ijns12030065

**Published:** 2026-08-10

**Authors:** Carolyn Mazariego, Mahitha Ramanathan, Deborah A. Johnston, Zhicheng Li, Brittany C. McGill, Marina Okamura, Lauren Kelada, Ilona Juraskova, Claire E. Wakefield, Natalie Taylor

**Affiliations:** 1 Implementation to Impact (i2i), School of Population Health, Faculty of Medicine and Health, UNSW Sydney, Kensington, NSW 2052, Australia; c.mazariego@unsw.edu.au (C.M.); m.ramanathan@unsw.edu.au (M.R.); deborah.johnston@unsw.edu.au (D.A.J.); zhicheng.li@unsw.edu.au (Z.L.); 2 Behavioural Sciences Unit, School of Clinical Medicine, Discipline of Paediatrics and Child Health, UNSW Medicine and Health, UNSW Sydney, Kensington, NSW 2052, Australia; b.mcgill@unsw.edu.au (B.C.M.); l.kelada@unsw.edu.au (L.K.); clairew1@stanford.edu (C.E.W.); 3 Minderoo Children’s Comprehensive Cancer Centre, Sydney Children’s Hospital, Randwick, NSW 2031, Australia; 4 School of Psychology, The University of Sydney, Sydney, NSW 2006, Australia; marina.okamura@sydney.edu.au (M.O.); ilona.juraskova@sydney.edu.au (I.J.); 5 Division of Quality of Life and Pediatric Palliative Care, Department of Pediatrics, Stanford University and Stanford Medicine Children’s Health, Palo Alto, CA 94304, USA

**Keywords:** newborn screening, genomic newborn screening, informed consent, genomic testing, qualitative research

## Abstract

Informed consent is fundamental to Australian newborn bloodspot screening (NBS), but emerging genomic screening technologies pose new challenges to clinical care and research. Optimizing consent processes is necessary to support ethical practice and maintain public trust as NBS evolves. This study aimed to identify gaps and opportunities to improve NBS consent processes in Queensland, Australia, while also exploring preliminary insights into the evolving complexity of consent in the context of genomic NBS (gNBS) and NBS-related research. A qualitative study design was used, with two facilitated interest-holder workshops (a total of 12 h) involving 86 participants (healthcare professionals, policy-makers, researchers, genomics experts, and consumer representatives). Workshop 1 (*n* = 25) was held virtually, and Workshop 2 (*n* = 61) was held in person. Thematic analysis was used to identify practical recommendations and ethical considerations. Participants identified three priority domains for improving consent in Queensland’s NBS program: (1) revision of the Guthrie Card and consent statement, (2) development of consistent, antenatal information resources across healthcare providers, and (3) standardized consent delivery training for healthcare staff. Discussions also highlighted tensions around information requirements for informed consent, revealing growing complexities regarding layered consent models. While recommendations on research consent were not fully developed at the workshop, insights highlighted the growing complexity and divergence of views on layered consent models. Findings suggest that improving NBS consent requires both operational reform and reassessment of ethical standards, alongside broader interest-holder engagement and feasible, scalable models also suited to genomic technologies. A nationally consistent framework is needed.

## 1. Introduction

Newborn bloodspot screening (NBS) is a critical public health intervention that enables the early detection and treatment of serious genetic and congenital genetic conditions in asymptomatic infants [1]. Screening is conducted by collecting a newborn’s heel-prick blood sample on a Guthrie Card, specifically designed for NBS sample collection, before testing it on a range of conditions where early intervention may reduce disability or death. Some countries offering NBS, including the United States, have integrated NBS into routine newborn care and do not actively seek consent [2]. In Australia, however, explicit parental consent is required for participation in NBS across five programs providing national coverage. Australian NBS uptake is approximately 99% of all live births [3] with a treatable condition identified in approximately 1 in 1000 screened newborns [3].

In 2018, Australia introduced a National Policy Framework for national alignment across the five NBS programs [4], which outlines core program elements across states and territories, including recommendations on the timing and methods of information provision for consent. Informed parental consent is a foundational component of NBS in Australia, with parents required to provide either written or verbal consent prior to bloodspot collection [4]. Despite this framework, consent processes continue to vary substantially across jurisdictions. Some Australian states require consent processes to include separate consent for testing and storage, explicit consent for research participation, a refusal of screening form, information on Guthrie Card storage, and access to information about commonly diagnosed conditions -while others have differing approaches [5]. For example, consent processes may include information on sample storage and diagnosed conditions but do not require separate consent for testing and storage or research participation [6]. In a small number of states, consent processes provide information on commonly diagnosed conditions and include a refusal of screening form, but omit options for consent to secondary data use, such as for research or separate consent for testing and data storage [7]. As a result, procedures for conducting research using previously collected samples are not clearly understood across settings.

**Figure 1:**
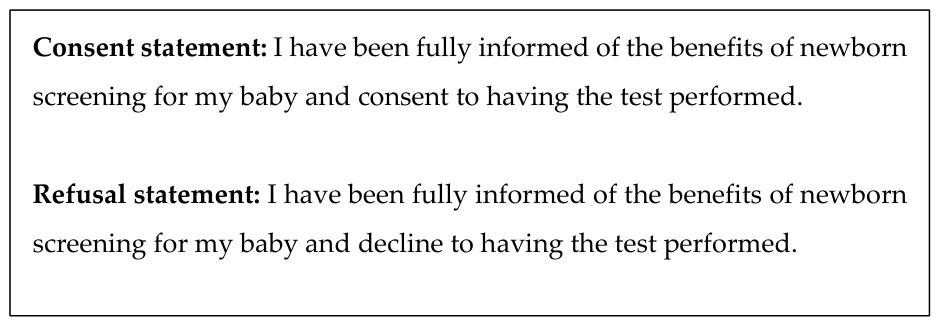
Example consent and refusal statements on Guthrie Card.

In the Queensland NBS program, which screens infants born in Queensland and the majority of the Northern Territory, parental consent is requested by the bloodspot collector immediately prior to sample collection. This is typically undertaken by a nurse or midwife who has received education and training regarding sample collection, including the requirement to request and document consent in advance. Consent or refusal is recorded on the Guthrie Card (example wording of Guthrie Card in use at the time of this study is presented in Figure 1). The mother may complete and sign the Guthrie Card; however, consent is often provided verbally [8].

To strengthen the state’s NBS program, Queensland published the Newborn Bloodspot Screening Strategic Framework in 2023 [8]. The framework identifies opportunities for program optimization whilst recognizing the complexity of delivering NBS across diverse healthcare settings, highlighting the need for alignment to ensure programs continue to meet the needs of families. Key areas for improvement identified in the framework include ensuring families provide informed consent, improving access to plain language information particularly for priority populations, and developing statewide guidelines and quality standards. Consistent and transparent consent processes are essential to support equitable access to information and informed decision-making [9]. At the time this work was conducted, and to the best of our knowledge at the time of writing, there is limited formal guidance to support consideration of research conducted within the Queensland NBS program.

Advances in genomic technologies are further complicating consent processes, as the boundaries between standard NBS, genomic NBS (gNBS), and genomic NBS-related research (gNBS-R) become increasingly blurred. Traditional NBS relies on biochemical markers to detect a limited number of conditions, whereas gNBS has the potential to identify hundreds of conditions, raising new considerations for consent processes. Concurrently, there is growing interest in integrating research into NBS programs, including the use of stored bloodspots and prospective genomic sequencing within clinical trials [10]. These developments introduce additional consent complexities, particularly when research participation is layered onto routine screening.

Evidence suggests that healthcare professionals support the inclusion of genomic sequencing in NBS, and Australian parents have expressed willingness to consider its integration [10,11]. Reflecting this momentum, the Australian Genomics Health Futures Mission and the National Health and Medical Research Council allocated over AUD $66m in 2022 to research exploring genomic sequencing in NBS [12]. The Australian Government has also committed AUD $107.3m between 2022 and 2028 to expand NBS and improve national consistency [13]. Public discourse has emphasized the need to modify existing consent processes, particularly regarding consent timing and workforce capability to support genomic screening [14,15].

Against this backdrop, this study aimed to develop recommendations on how to identify ways to optimize routine NBS consent and consider how consent processes might be future-proofed in response to emerging research and technological advances. Using a structured workshop approach [16], the study engaged key interest-holders, defined as groups directly involved in or affected by health-related decision-making [17], to examine current consent challenges, identify opportunities to enhance transparency and information provision, and develop recommendations to support both routine screening and research within evolving NBS programs.

## 2. Methods

### 2.1. Study Design

Two consensus workshops were conducted with subject matter experts involved in the NBS consent process and broader program implementation, employing a participatory qualitative research design. A participatory qualitative research design is a qualitative approach in which the people who are affected by the issue being studied are actively involved in the research process (e.g., through interviews or focus groups), rather than simply serving as participants [18]. The primary focus of the workshops was on Queensland’s NBS consent processes; but insights from individuals with exposure to consent processes within and outside the state were deemed crucial to ensure insights were nationally relevant, so representation from other Australian states and territories was sought.

Workshop 1 was conducted virtually via Microsoft Teams in March 2024, while Workshop 2 was held in person in Brisbane, Queensland, in May 2024. 

### 2.2. Participants

Participants were eligible if they had subject matter expertise in Australian NBS consent and were able to attend at least one of the two workshops. Participants were recruited through a combination of targeted invitations via professional networks of the project steering group, publicly available information (e.g., state and territory NBS program websites), and snowball sampling, where invited participants recommended additional potential attendees. The project steering group included researchers in population health, genomics and psychology, clinicians and service providers involved in newborn screening, and members of rare disease advocacy groups, with representation from Pathology Queensland, Queensland Health, and UNSW.

Written informed consent was obtained before participation, and verbal consent was reconfirmed prior to the recording of each workshop.

### 2.3. Data Collection

In preparation for the workshops, a summary table of the five screening programs in Australia and their inclusions were created to generate discussion around strategies for national coordination (Table 1). Each workshop followed a structured format combining presentations from invited speakers with small-group breakout discussions facilitated by implementation science researchers from UNSW. Five facilitators (CM, NT, BM, CW, ZL) were briefed ahead of the workshops as to the aims and specific activities required. All are qualitative and/or implementation science researchers with previous experience of workshop facilitation.

Each workshop was audio-recorded, and transcripts were generated for analysis. Within the workshop, there were smaller randomly assigned breakout groups. Breakout group discussions were not recorded but facilitators were asked to take notes and present back to the whole group on what was discussed in smaller groups. Results were reported in line with the COREQ (Consolidated Criteria for Reporting Qualitative Research) a checklist designed to improve the clarity and completeness of reporting qualitative studies, including focus groups (Supplementary Materials). Participants’ input was sought on predetermined discussion topics: identification of gaps and opportunities for improving consent in Queensland’s NBS program; potential solutions and their feasibility; considerations for obtaining consent for genomic testing in an expanded NBS framework.

#### Workshop Activities

**Figure 2:**
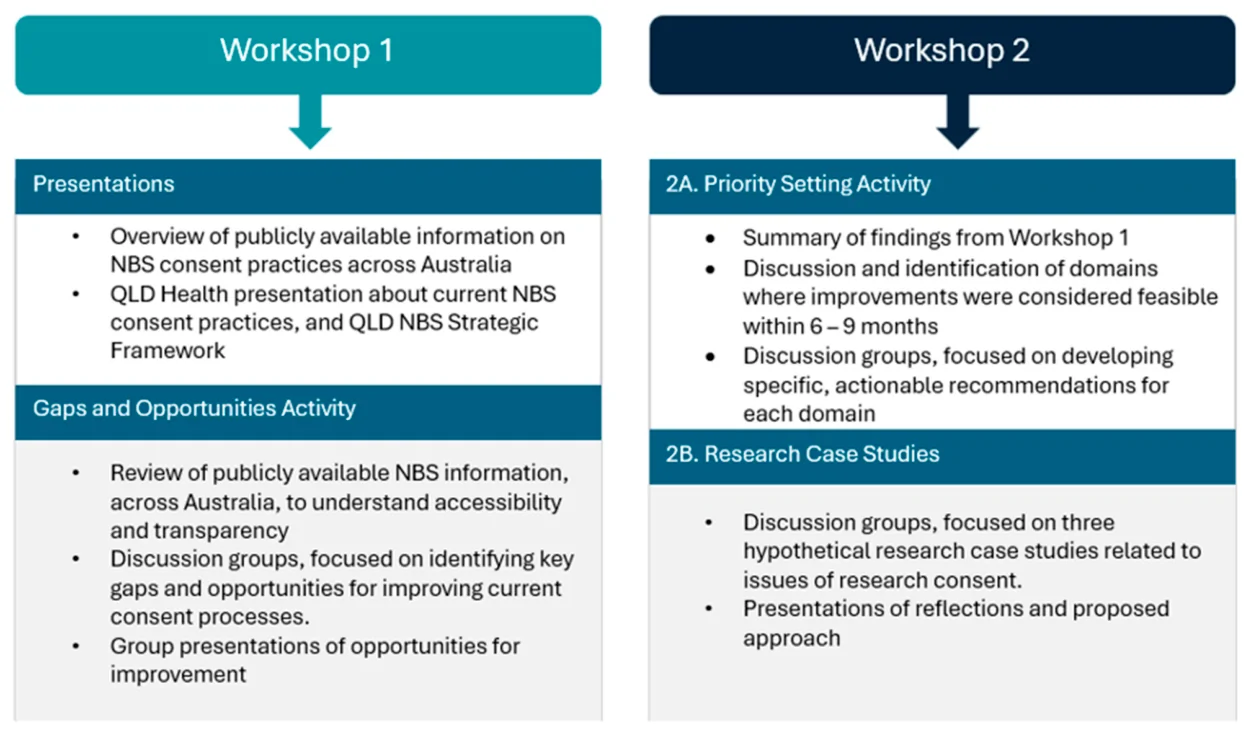
Activities included in workshops. Abbreviations: NBS: Newborn Bloodspot Screening; QLD: Queensland.

Figure 2 details components of each workshop. In both workshops, groups discussed ethical considerations, system integration, and consent design, before reporting key reflections and proposed approaches back to the plenary session for cross-case comparison and discussion.

### 2.4. Data Analysis

After Workshop 1, an inductive content analysis of the discussions was undertaken to identify and categorize the key domains in which improvements were considered necessary. Inductive content analysis is a qualitative analytical approach used when there is limited existing theory or prior knowledge about a phenomenon. Rather than applying a pre-existing coding framework, categories and themes are derived directly from the data through a systematic process of coding and abstraction (conducted by CM and MR) [19]. Analysis from Workshop 1 aimed to identify areas for improvement of NBS consent processes, which were then taken to Workshop 2, where recommendations on how to address these challenges would be discussed. Inductive content analysis of Workshop 2 transcripts then identified recommendations on how to improve consent processes for NBS across the challenges identified in Workshop 1. Workshop 2 also presented research case studies where discussions focused on potential pathways to seek, obtain, or waive consent were considered appropriate.

## 3. Results

**Figure 3:**
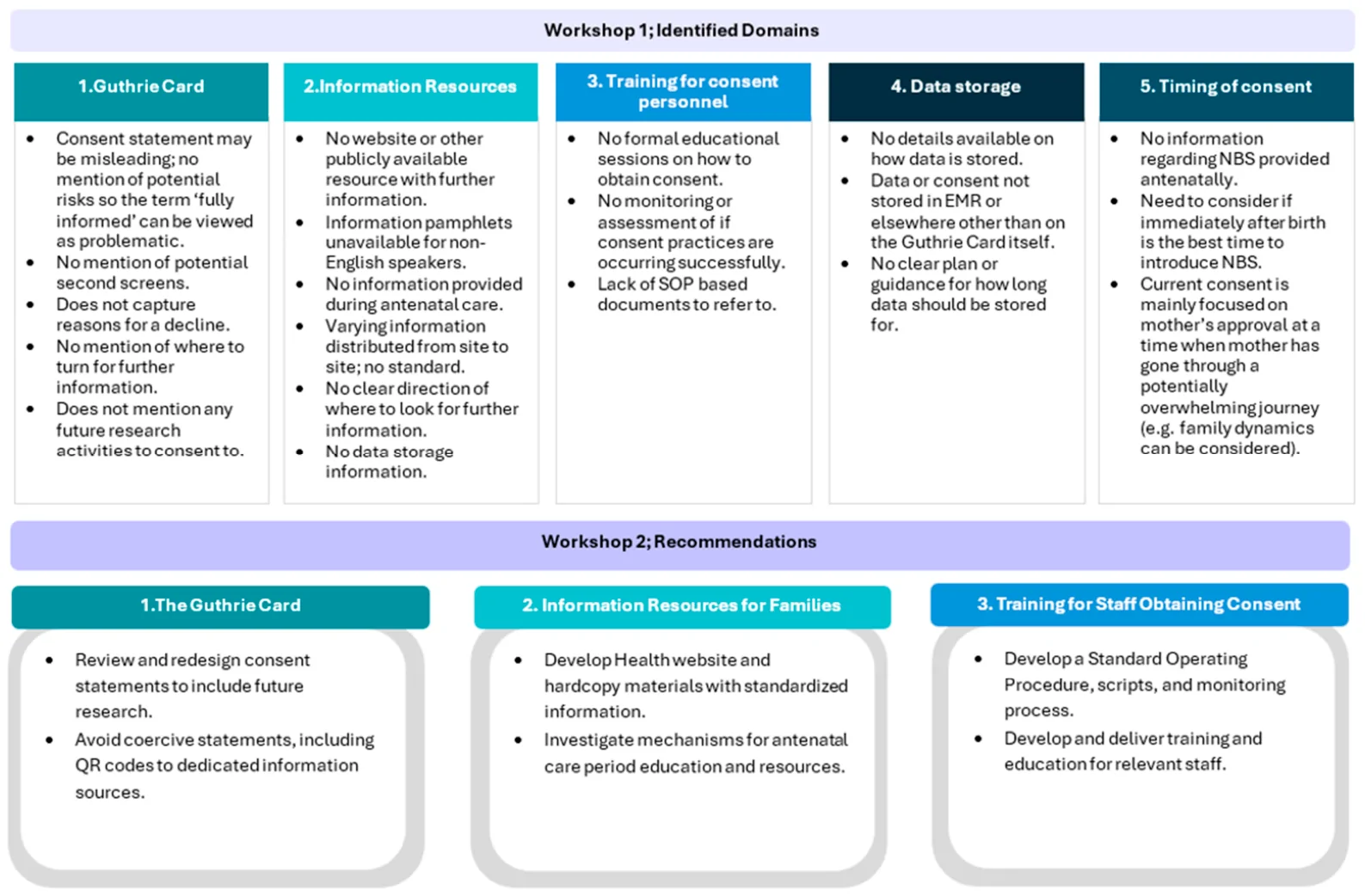
Identified domains for potential opportunities for improving consent practices. Abbreviations: EMR: Electronic Medical Record; NBS: Newborn Bloodspot Screening; Qld: Queensland; SOP: Standard Operating Procedure.

A total of 86 individuals participated across the two workshops. Workshop 1 was attended by 25 participants, including 10 healthcare professionals (midwives, NBS program staff, and pediatric care providers), 5 government and policy representatives, 8 academic researchers, and 2 consumer representatives (people with lived experience of participating in NBS [parents]). Workshop 2 included 72 participants, comprising 32 healthcare professionals, 21 government and policy representatives, 15 academic researchers, 4 consumer representatives, and 5 patient advocacy group representatives. Invitations for Workshop 2 were distributed following Workshop 1. The second workshop included both returning and newly recruited participants, resulting in a larger and more diverse group of stakeholders. Figure 3 outlines key themes and opportunities for improvement identified throughout the two workshops.

### 3.1. Workshop 1: Identification of Key Domains and Challenges for Current NBS Consent Practices

Five key domains where gaps and challenges in current NBS consent were identified from Workshop 1. Table 2 provides supporting quotes:

*Domain 1. The Guthrie Card (NBS consent form):* Participants raised concerns about the Guthrie Card and the clarity of its consent statement. Participants perceived that the existing consent and refusal statements may only highlight the benefits of screening without mentioning potential risks. There was broad agreement that this omission could impact whether parents are fully informed of not only the benefits, but also the risks, when providing consent. Participants also stated that the Guthrie Card does not mention the possibility of a second screen, nor does it capture reasons for declining consent (Table 2, quotes 1–3).
*Domain 2. Information resources for families:* Regarding information resources for families, participants highlighted a perceived lack of accessible and standardized information about NBS. They noted that they were not aware of any publicly available website with official state-wide NBS information, nor information tailored to culturally and linguistically diverse groups. Participants expressed concerns that parents may therefore have limited exposure to information about NBS during antenatal care, leaving them with little time to process information and make informed decisions. They also noted potential variability across hospitals in how NBS information is provided, which may have led to inconsistencies in parental understanding. A recurring issue raised by participants was that parents who seek further information would have no clear guidance on where to find reliable resources (Table 2, quotes 4 and 5).
*Domain 3. Training for personnel obtaining consent:* The training of staff to obtain consent was identified as an opportunity for improvement. Participants expressed that to their knowledge, there are no detailed formal education sessions specific to consent for healthcare personnel responsible for discussing NBS consent with parents. Additionally, the absence of standard operating procedures (SOPs) meant that staff may lack a clear framework for guiding consent discussions. Discussion around monitoring or assessment of consent practices and the difficulties in ensuring that all parents receive accurate and comprehensive information before making their decision, was a notable topic (Table 2, quote 6).
*Domain 4. NBS data storage:* Participants felt that the NBS data storage management processes could be clearer and more transparent. Participants detailed that consent information and screening data were not stored in electronic medical records (EMRs) or any centralized database, aside from being recorded on the Guthrie Card itself. This was perceived to create potential uncertainty regarding how long access is regulated. Some interest-holders raised concerns about data security and governance, particularly in light of increasing digital health initiatives (Table 2, quote 7).
*Domain 5. Timing of consent:* There was a perceived mismatch between recommended timing of information provision and current practice. Given the emotional and physical demands of childbirth, some participants questioned whether the immediate postnatal period was the optimal time to introduce NBS consent discussions. While consent can be obtained from either parent, participants observed that the birthing parent’s approval is often sought when they may feel overwhelmed post-childbirth, potentially affecting decision-making (Table 2, quote 8).

### 3.2. Workshop 2: Prioritization and Development of Recommendations for Current NBS

Participants refined the five domains derived from the synthesis of Workshop 1 into three priority areas based on feasibility for short-term improvement action areas; the three domains of focused discussion included: the Guthrie Card, information resources for parents, and training for staff obtaining informed consent.

#### 3.2.1. Priority Area 1: The Guthrie Card

Expanding the consent statement: Participants expressed concerns that the current consent statement, “*I have been fully informed of the benefits of newborn screening*”, could appear unbalanced. However, they also cautioned that excessive risk information might discourage participation (Table 2, quote 9).
Incorporating research participation: Participants recommended implementing an opt-in/opt-out research statement on the Guthrie Card to allow parents to choose whether to participate in de-identified or anonymous research (Table 2, quote 10).
Providing further information: To ensure accessibility, participants suggested including a QR code linking to an online resource with detailed, up-to-date information about NBS for the state. However, concerns were raised about internet access in rural areas (Table 2, quote 11).

#### 3.2.2. Priority Area 2: Information Resources for Families

Statewide Consistency: Participants emphasized the need for standardized, easily accessible information about NBS and pathways following a positive screen. A proposed solution was to develop a state-wide NBS website hosted by Queensland Health. The website should be user-friendly and visually engaging, offer multilingual content, include a video demonstration of the screening process, and be linked to the Guthrie Card via a QR code (Table 2, quotes 12 and 13)
Timing of Consent Discussions: Participants highlighted that discussing NBS consent immediately postpartum may be overwhelming for parents, potentially leading to hesitation or refusal. Recommendations included introducing a clearer “consent primer” during antenatal care, integrating NBS information into routine pregnancy screening, and linking NBS education to the birth-parent’s pregnancy health record to facilitate discussion with healthcare providers (Table 2, quotes 14 and 15).

#### 3.2.3. Priority Area 3 Training for Staff Obtaining Consent

Formal Education Sessions: Participants recommended standardized training for healthcare staff involved in NBS consent, ensuring that all professionals provide consistent information. Suggested approaches included the development of a script for obtaining consent, training for consenting personnel with plain-language information on the Guthrie Card and a website that parents can readily comprehend, creating SOPs for consent.
Standardized Processes: Participants further suggested that Queensland Health or Queensland Clinical Guidelines could oversee SOP development, ensuring uniform consent practices across the state (Table 2, quote 17). This might include monitoring and assessment efforts to ensure consent practices are effectively implemented (Table 2, quotes 18).
Monitoring and Evaluation: It was further recommended that state-based approaches to consent should include the establishment of a quality assurance framework, discussion during the antenatal period from established care teams, and an auditing process of consent practices to assess effectiveness and identify areas for improvement (Table 3, quote 19).

### 3.3. Workshop 2: Consent for Research Within/Alongside the NBS Program

Participants explored ethical and logistical considerations related to potential research conducted within or alongside the newborn screening (NBS) program. Using three hypothetical case studies, groups discussed how the proposed research could be operationalized and what type of consent would be recommended. The discussion’s main themes and notable tensions are summarized in Table 3.

**Table 3:** Research case studies presented and discussed—main themes and notable tensions.

	Research Case Study 1: *Exploring Parental Experiences Following Positive NBS Results*	Research Case Study 2: *Retrospective Analysis of Archived Bloodspot Samples Using New Screening Technologies*	Research Case Study 3: *Prospective Implementation of Targeted Genetic Sequencing in Newborn Screening*
**Brief description**	A qualitative study involving interviews with parents of infants who received a positive NBS result, aiming to understand their experiences and identify improvements to the NBS process.	A retrospective research study using stored Guthrie Cards to test new genomic technologies and compare results to the standard NBS panel.	Prospective study introducing targeted genetic sequencing alongside routine NBS, with results returned to families as part of a pilot program evaluating implementation and clinical utility.
**Main themes**	Nature of the Research		
	Participants generally agreed this was low-risk, observational research but warranted special ethical consideration due to the emotional vulnerability of participants.	Identified as secondary use of identifiable samples, raising ethical concerns about consent adequacy and public trust.	Blurred lines between clinical care and research; perceived as a hybrid model (pilot testing within standard care) (Table 3, quotes 27 and 28).
	Consent Approach		
	Recommendation for opt-in, written or verbal consent, with strong emphasis on timing, psychosocial support, and clear separation from clinical care (Table 3, quotes 20, 21, and 22).	Divided opinions: Some argued for broad, opt-in consent for future research at the time of NBS (Table 3, quote 26). Others suggested re-consenting may be necessary depending on how identifiable the samples are and the sensitivity of the genomic data (Table 3, quotes 23–25).	Clear support for tiered, opt-in consent with a separate pathway from routine NBS. Must include detailed pre-test counseling and follow-up support.
	System Implications		
	Need for clear protocols for approaching families, with some participants suggesting this could be embedded into follow-up workflows after a positive screen result.	Strong need for transparent communication with the public and a governance framework for access and oversight.	Concerns about health literacy, equity, and clinical workforce capacity to deliver meaningful consent at scale (Table 3, quote 29).
**Notable tensions**	Ethical sensitivity vs. practical timing (when and how to approach families). Concern about re-traumatizing parents vs. the value of capturing lived experience.	Practicality of re-consent vs. ethical standards. Broad consent models vs. specific informed consent.	Divergence on whether this should be positioned as research or early implementation. Whether consent can be truly informed if there is limited public understanding of genomics.
**Cross-cutting themes**	The need for tiered and flexible consent frameworks that distinguish between routine screening, optional opportunities to receive expanded screening and research. Persistent ethical tensions between feasibility, autonomy, and program integrity. The importance of system-wide supports (training, infrastructure, governance) to operationalize meaningful consent in increasingly complex NBS systems.		

Abbreviations: NBS: Newborn Bloodspot Screening.

## 4. Discussion

This study provides one of the first interest-holder driven, system-level examinations of NBS processes in Australia conducted at a time when emerging genomic technologies have the potential to fundamentally reshape both the ethical and operational foundations of screening programs. Participants identified several gaps in current NBS consent processes in the state, including limitations in the Guthrie Card consent statement, inconsistent information resources for families, inadequate staff training, unclear data storage practices, and concerns about the timing of consent discussions. Collectively, these issues may compromise informed decision-making by parents and highlight the need for a more transparent, standardized, and supportive consent approach. Strong consensus was reached on several practical priorities, including the need for earlier consent discussions, ideally during the antenatal period; centralized, accessible information such as an NBS website; and standardized training for health professionals responsible for providing consent information. These recommendations reflect a shared view that consent should be treated as a process, rather than a single point-in-time interaction. Beyond these operational improvements, participants highlighted increasing complexity associated with genomic NBS and genomic NBS-related research. Although the workshops did not generate formal recommendations in this area, discussions highlighted key ethical tensions and operational challenges, including whether existing systems could accommodate layered consent models for data storage, secondary use and research, and how much information is sufficient to facilitate autonomy without overwhelming or deterring parents.

The tensions identified in the current Australian system capacities to expand consent models and adequately support parental decision-making align with the international literature documenting similar challenges in standard NBS consent processes. Studies from the United Kingdom, United States, and Canada have highlighted the importance of providing NBS information during pregnancy, as parents often report feeling unprepared to make consent decisions in the immediate postnatal period [20,21,22]. Prior research has also identified limited transparency regarding bloodspot storage and secondary use, with concerns that parents may not fully understand how their child’s samples or data could be used for research [2,4]. Recommendations for tiered consent models are consistent with global discussions aimed at enhancing parental autonomy while preserving program effectiveness [20]. In addition, the importance of training and standardization for consent providers has been widely recognized as critical to ensuring consistent and informed decision-making across healthcare settings [4,8].

One of the most salient insights from Workshop 2 was the need to balance the provision of sufficient information to enable informed decision-making for valid consent without the risk of information overload. Designing and delivering antenatal NBS information resources for parents requires careful consideration to ensure such information is both meaningfully detailed while being manageable for parents in terms of accessibility, English language and health literacy burden. Such education resources must also suit the varied linguistic and cultural needs of Australia’s diverse, multi-cultural populations, including co-design and consultation with parents and community representatives where possible. Although the study sought to explore consent challenges related to genomic NBS and research, discussions in Workshop 2 only began to address the tip of the iceberg of these issues. Participants recognized the complexity of integrating research consent into routine screening and expressed divergent views on appropriate models. While some supported layered, opt-in approaches distinguishing clinical care from research, others raised concerns about feasibility and the potential impact on screening uptake. These discussions reinforced that consent within NBS is context-dependent and must be embedded within systems in ways that are both ethically acceptable and operationally viable.

The themes raised during the workshops reflect the inherent ethical complexity of consent within a highly valued and effective public health program. NBS improves outcomes for tens of thousands of children each year and is widely recognized as a cornerstone of preventive healthcare [23]. As programs evolve to incorporate whole genome sequencing and expanded research initiatives, maintaining high participation rates while ensuring meaningful informed consent becomes increasingly critical. Workshop discussions revealed clear tensions between preserving the benefits of the program and ensuring families are adequately informed. Debates regarding opt-in versus opt-out consent models highlighted the need for clarity and consistency, and posed the call for national leadership in this space. Collectively, these challenges point to an urgent need for coordinated action in Australia across NBS programs, to guide implementation and establish clear ethical and operational principles. 

## 5. Strengths and Limitations

This study benefited from broad interest-holder engagement across healthcare, policy, research, and consumer advocacy sectors, providing rich insights into the ethical and operational challenges of informed consent in NBS. However, several limitations should be acknowledged. First, although participants represented diverse professional backgrounds, the recording for Workshop 2 did not specify which interest-holder was speaking at any moment, making it impossible to disaggregate perspectives by role.

Second, while the workshops did include consumer perspectives of parents who consented to newborn screening (*n* = 3) and representation from rare disease advocacy organizations, perspectives from First Nations communities, culturally and linguistically diverse populations, and rural and remote communities were not comprehensively represented and should be prioritized in future research. Finally, although the workshops addressed both routine and research-related consent, the discussion of research consent, particularly in genomic screening and the use of archived samples, was not sufficiently detailed to support specific recommendations. This reflects the inherent complexity of the topic, including unresolved questions around data governance and consent models. Nevertheless, the discussions provided valuable preliminary insights into divergent perspectives that warrant further investigation.

## 6. Future Directions and Conclusions

The findings from this study provide a foundation for developing an actionable roadmap to improve informed consent processes within NBS programs, while also informing broader national discussions as genomic NBS continues to emerge. The priority areas identified via this research study are expected to be further developed into actionable recommendations for consent improvement via an expert working group. Future work should prioritize piloting proposed improvements to routine consent, including antenatal education strategies and statewide information systems, and evaluating their impact on informed decision-making and equity of participation. Further research is also needed to develop effective methods for building consensus on ethically sensitive issues with legal and operational implications, particularly where interest-holder views may diverge.

## Figures and Tables

**Table 1:** A comparison of consent features across Australian Newborn Bloodspot Screening Programs (current as of 2024 and used to guide workshop discussions).

Features of Information Provided and/or Guthrie Card	Number of States/Territories Representing This Information
Separate consent for testing and storage of data	2/8
Separate consent for research	3/8
Refusal of screening form	7/8
Storage of screening card explained	7/8
Websites listed for more information	4/8
Most commonly diagnosed conditions listed	8/8

**Table 2:** Example quotes from workshop participants.

**Workshop 1: Identification of key domains and challenges for current NBS consent practices**	
Domain 1. The Guthrie Card (NBS consent form)	“The current (consent) statement only talks about the benefits of the screen. Informed consent usually requires a discussion about both the risks and benefits. The lack of this makes the statement appear coercive.” (Genomics Expert)
	“Explicit consent is the way of the world. It is the right of the parent and the child to be informed.” (Healthcare Professional)
	“What’s the line between coercion and informed consent? How do we ensure we are not losing numbers but sharing the right amount of information?” (Academic Researcher)
Domain 2. Information resources for families	“There is a gap in resources, especially for First Nations (Indigenous Australian populations) and culturally and linguistically diverse communities. Having this information gap prevents us gaining fully informed consent.” (Genomics Expert)
	“You can’t give people everything, it’s up to them to do the research. It’s more about making the information easy and accessible to as many people we can.” (Government and Policy Representative)
Domain 3. Training for personnel obtaining consent	“Currently, the extent of ‘informed consent’ depends on the health professional who takes the blood (sample). Some of them may not have the knowledge about all the conditions tested and their implications. There needs to be a balance on how much information they should give, as too much information can create extra burden.” (Healthcare Professional)
Domain 4. NBS data storage	“Nowhere on the Guthrie Card does it say anything about how long data will be stored for. At times, the Guthrie Card is all people see before making a decision—so you have to ask yourself, ‘are people really understanding what they are consenting to?’ (Genomics Expert)
Domain 5. Timing of consent	“Post-delivery, a new mother has just had a baby, and she is exhausted. It’s not the right timing to be gaining consent for anything, because the mother is not going to be in the right frame of mind to provide consent.” (Healthcare Professional)
**Workshop 2A: Priority areas and recommendation development for Queensland NBS consent**	
Priority area 1. The Guthrie Card	
Expanding the consent statement	“(It should include) ‘I have been provided with information on the newborn screening program and have had the opportunity to ask questions’. ‘I provide my consent for my child to be screened’.”
Incorporating research participation	“Potentially adding a statement that says ‘I consent to be contacted about research’ would be beneficial, because sometimes an opt-in tick isn’t entirely representative of consent to research activities.”
Providing further information	“A QR code or spelling out the link to a website would be ideal, as the term ‘fully’ (on the current card) is too intense of a term to describe the current information given. It is sufficient information, but we should also say ‘If you would like to know more, here’s the link to a website’.”
Priority area 2. Information resources for families	
Statewide consistency	“A Queensland Health hosted website could link to other resources, but should centralize information specific to the state’s NBS program and the pathways that can flow for families depending on their NBS outcome.”
	“A NBS video (on a website) would allow parents to observe what a NBS test looks like, so they can expect their provider to follow the same steps during their test.”
Timing of consent discussions	“Information sharing should happen in antenatal care. Pregnancy is a wonderful time for screening. We screen women with ultrasounds and blood tests, so why wouldn’t we introduce another type of screening?”
	“There should be a real onus on midwives to provide and disseminate that (NBS) information, because midwives are educators; that’s their most important role.”
	“This is just more screening, like an ultrasound, you can choose to do it, and if you choose to do it, these are the best practice outcomes.”
Priority area 3. Training for staff obtaining consent	
Formal education sessions	“…a SOP could be developed at the higher levels of Queensland Health, but coordinated through Queensland Clinical Guidelines (a state government organization). It should be a living document, given that NBS practices are likely to change with advances in technology.”
	“If we have this training in place, there needs to be a record of training or accreditation that needs to be maintained to provide quality assurance. This could be through a midwife manager monitoring staff, or patient satisfaction on patient surveys.”
	“The engagement and upskilling of GP’s is important; whilst they may not do the actual heel pricks, it’s important that they know how to answer questions and where to send parents for further information.”
**Workshop 2B: Consent for *research* within/alongside the NBS Program**	
Case Study 1: Research to conduct qualitative interviews with parents of screen-positive infants	“There’s a need to ensure psychological support is in place, including researchers doing interviews having been trained in psychology or genetic counselling. Have the right people do the interviews, include follow-up, and tailor your approach based on if you’re talking to a true or false positive case.”
	“The timing of the parental interview may impact results. In the case of a prospective study, you would be interviewing parents in a potentially emotional period whilst the diagnosis is still fresh in their mind, and this bias results accordingly.”
	“In the case of a prospective study, a multi-stage consent process would be best. Parents should provide consent before they undertake the test but again be asked to provide consent to partake in the (research) interview once they have received the positive result.”
Case Study 2: Research to conduct retrospective analysis of stored bloodspots using genomic technologies	“In the case of a retrospective study, there are logistical questions in how you go about identifying and contacting cases. There may be recall problems, relationships issues that make it difficult to access parents easily.”
	“The process of collecting and re-punching archived Guthrie Cards alongside reviewing and resorting consent would be a burden on lab staff. Grant applications (funding research) should have incorporated this and have money to support this.”
	“What conditions are going to be tested for? Ideally, you should test with the same conditions so as to not introduce new tests or consent processes. If you wish to go broader and test for new conditions, that will require new consent, because what are you going to do if you find something? Do you have processes in place to contact families and people? Are care pathways established? You won’t be able to keep these de-identified in that case.”
	“On the assumption of testing for conditions already on the program, we thought it would need a reconsent, because what happens if something is not picked up through the biochemical screen that is picked up through genomic screening? Need to consider how this will be fed back to parents.”
Case Study 3: Research to conduct prospective implementation of targeted genetic sequencing	“On the consent process, there needs to be general consent for NBS…and an option for access to talk about the experience and ask further questions. There should also be an option for people doing consent to go to a space to access more information to support parents. We don’t want people to not take up newborn screening because the consent process is too complicated. This should also be supplemented with a standardized script, so everyone taking consent is saying the same thing.”
	“There needs to be more consideration of and communication to families choosing not to complete certain types of screening, and what this could mean for them down the track.”
	“There are some concerns with burdening parents with the technology, and the language we use around genomic technology. The term genetic, in the case of a positive diagnosis, can become laden with blame and guilt. There is a risk of overcomplicating NBS.”

Abbreviations: NBS: Newborn Bloodspot Screening. Footnote: Due to the nature of audio recording and summarized sharing of feedback from the in-person workshop (workshop 2), quotes could not be attributed to a particular participant at the workshop as they are derived from summary statements of group work/thought within group discussions.

## Data Availability

The datasets generated and/or analyzed during the current study are available from the corresponding author on reasonable request.

## Supplementary Materials

The following are available online at https://www.mdpi.com/article/10.3390/ijns12030065/s1.
