# Supplementary material for: Optimizing Informed Consent for Australian Newborn Bloodspot Screening and Research: Consensus Workshop Insights and Recommendations
Source: Int J Neonatal Screen. 2026 Aug 10;12(3):65. doi: 10.3390/ijns12030065 (PMC13510761; doi:10.3390/ijns12030065)
Supplement: Supplementary file 1 [file IJNS-12-00065-s001.zip › IJNS-4327223-supplementary.pdf]

No	Item	Guide questions/description	Included?
**Domain 1: Research team and reflexivity**			
Personal Characteristics			
1.	Interviewer/facilitator	Which author/s conducted the interview or focus group?	Y, Methods
2.	Credentials	What were the researcher's credentials? *E.g. PhD, MD*	Y, Title page
3.	Occupation	What was their occupation at the time of the study?	Y, Methods
4.	Gender	Was the researcher male or female?	N
5.	Experience and training	What experience or training did the researcher have?	Y, Methods
Relationship with participants			
6.	Relationship established	Was a relationship established prior to study commencement?	N
7.	Participant knowledge of the interviewer	What did the participants know about the researcher? e.g. *personal goals, reasons for doing the research*	N, we specify that workshops are all invited experts in the field.
8.	Interviewer characteristics	What characteristics were reported about the interviewer/facilitator? e.g. *Bias, assumptions, reasons and interests in the research topic*	Y, Methods (implementation science team from UNSW)
**Domain 2: study design**			
Theoretical framework			
9.	Methodological orientation and Theory	What methodological orientation was stated to underpin the study? *e.g. grounded theory, discourse analysis, ethnography, phenomenology, content analysis*	Y, methods – thematic analysis
Participant selection			
10.	Sampling	How were participants selected? *e.g. purposive, convenience, consecutive, snowball*	Y, Methods

11.	Method of approach	How were participants approached? e.*g. face-to-face, telephone, mail, email*	Y, Methods
12.	Sample size	How many participants were in the study?	Y, methods & results
13.	Non-participation	How many people refused to participate or dropped out? Reasons?	Y, methods
Setting			
14.	Setting of data collection	Where was the data collected? e.*g. home, clinic, workplace*	Y, Methods
15.	Presence of non-participants	Was anyone else present besides the participants and researchers?	N
16.	Description of sample	What are the important characteristics of the sample? *e.g. demographic data, date*	Y, methods
Data collection			
17.	Interview guide	Were questions, prompts, guides provided by the authors? Was it pilot tested?	Y, methods & results
18.	Repeat interviews	Were repeat interviews carried out? If yes, how many?	Y, methods – repeat workshop, not interviews
19.	Audio/visual recording	Did the research use audio or visual recording to collect the data?	Y, methods
20.	Field notes	Were field notes made during and/or after the interview or focus group?	Y, methods
21.	Duration	What was the duration of the interviews or focus group?	Y, results
22.	Data saturation	Was data saturation discussed?	Y, methods
23.	Transcripts returned	Were transcripts returned to participants for comment and/or correction?	N
**Domain 3: analysis and findings**			
Data analysis			

24.	Number of data coders	How many data coders coded the data?	Y, methods
25.	Description of the coding tree	Did authors provide a description of the coding tree?	Y, methods & results
26.	Derivation of themes	Were themes identified in advance or derived from the data?	Y, methods – derived from data
27.	Software	What software, if applicable, was used to manage the data?	Y, methods
28.	Participant checking	Did participants provide feedback on the findings?	Y, methods and results
Reporting			
29.	Quotations presented	Were participant quotations presented to illustrate the themes / findings? Was each quotation identified? e.*g.* *participant number*	Yes, results
30.	Data and findings consistent	Was there consistency between the data presented and the findings?	Yes, results and discussion
31.	Clarity of major themes	Were major themes clearly presented in the findings?	Yes, results
32.	Clarity of minor themes	Is there a description of diverse cases or discussion of minor themes?	Yes, results
